# Six-Year Survival and Early Failure Rate of 2918 Implants with Hydrophobic and Hydrophilic Enossal Surfaces

**DOI:** 10.3390/dj3010015

**Published:** 2015-02-05

**Authors:** Olivier Le Gac, Ueli Grunder

**Affiliations:** 1 Cabinet dentaire, 968, Avenue du Gl. Leclerc, Agen F-47000, France; 2 Praxis Grunder und Schneider, Dufourstrasse 7a, Zollikon-Zürich CH-8702, Switzerland; E-Mail: u.grunder@bluewin.ch

**Keywords:** dental implants, early failure rate, case series, enossal surface hydrophilic *vs.* hydrophobic

## Abstract

The aim of this chart review was to obtain an objective, quantitative assessment of the clinical performance of an implant line used in an implantological office setting. Implants with hydrophilic (INICELL) and hydrophobic (TST; both: Thommen Medical AG, Grenchen, Switzerland) enossal surfaces were compared and the cumulative implant survival rate was calculated. The data of 1063 patients that received 2918 implants (1337 INICELL, 1581 TST) was included. The average follow up time was 2.1 (1.1–5.4) years for INICELL and 4.5 (1.3–5.9) years for TST implants (Thommen Medical AG, Switzerland). In the reported period 7 implants with INICELL (0.5%) and 23 TST implants (1.5%) failed. This difference was statistically significant. The analysis of cases treated and followed up in a single implantological office for 6 years confirmed the very good clinical outcome that was achieved with both used implant lines. Within the limitations of this retrospective analysis, the overall early failure rate of the hydrophilic implants was significantly lower than that of hydrophobic implants. The use of hydrophilic implants allows the clinician to obtain less early failures, hence the interest of an up-to-date surface for the daily work of an implant practice.

## 1. Introduction

The reconstruction of missing teeth by Titanium dental implants is currently the gold standard in dental rehabilitation [1,2]. The clinical performance of Titanium implants has been documented for certain brands but currently there is a wide variety of dental implants with limited, or even no clinical documentation. Ever since the first studies showing the clinical interest of the peculiar affinity of bone tissue for titanium, the industry has invested a lot in order to improve the implant surfaces [3]. That is why, in the course of time, clinicians had at their disposal implants simply machined, then in the 80s implants with rough enossal surfaces, which resulted in an improved rate of osseointegration. Later on, the microstructured, or moderately rough, surfaces realized through sand-blasting and acid etching became the gold standard with failure rates as low as 1% [1]. Recent developments should even improve osseointegration, especially by rendering the surfaces to become hydrophilic. Such is the case of INICELL^®^ which is created immediately before implantation by exposing the standard, sand blasted and thermal-acid etched (TST) surface to the conditioning liquid and thus raising also the surface energy. This modification has no influence on surface roughness. The advantage of hydrophilic enossal surfaces is in the earlier osseointegration [4]. The performance of the surfaces was evaluated first mechanically (removal torques), histomorphometric analyses allowed to determine the rate of contact between bone and implant (BIC) assuring a good quality of osseointegration [4]. Last but not least, clinical studies observe the behavior of these implants loaded earlier and earlier, for instance after 3 weeks [5]. The clinician may wonder about the interest of his patient to use implants that can be loaded after 3 weeks when those loaded at 2 months work perfectly, unless the failure rate can be diminished and the marginal bone loss reduced.

The aim of this paper was to obtain the survival rate and objective assessment of the clinical performance of the selected implant line. A retrospective chart review was conducted with the aim to clarify whether the subjective good experience with the used Titanium implant line is supported by clinical results. Implants with hydrophilic (INICELL^®^) and hydrophobic (TST^®^) enossal surfaces were compared.

## 2. Materials and Methods

Titanium ELEMENT RC implants (cylindrical enossal shape, self-cutting threads, 1 mm polished collar for optimal esthetic results; Thommen Medical AG, Grenchen, Switzerland) have been used since 2007. The implants have a moderately rough enossal surface (sand-blasted-acid etched; TST). In 2010, the use of the same implant line with a super-hydrophilic surface (INICELL^®^; Thommen Medical, Grenchen, Switzerland) was started.

Owing to the retrospective data analysis, the patients have not been exposed to any additional risk; therefore, an Ethical Committee approval was not sought for. All measures have been taken in order not to disclose any patient personal data. Included are all patients that have received implants, no exclusion from the analysis was done, e.g., for known risk factors, such as smoking, findings in medical history, *etc.* The retrospective results represent the review of all consecutively placed implants in a specialized center for oral implantology. As for the surgical implant bed preparation technique, the manufacturer instructions have been followed. Patients that received both INICELL and TST implants underwent the following treatment protocols:

- immediate loading;
- immediate temporization with bone grafting, mainly for simultaneous and two-stage sinus lifting using DBBM (Bio-Oss, Geistlich, Switzerland) or β calcium triphosphate (TCP, CEROS, Thommen Medical AG, Grenchen, Switzerland);
- guided bone regeneration using either autogenous bone from the retromolar area or autologous bone (TBF, Mions, France). Augmentation surgery has been performed prior to the implant placement. The bone healing time was 4 months when autogenous bone was used, 5 months when patients received allogeneic bone, and 6 months or more with the bovine bone.

Based on the recorded implant survival, the cumulative implant survival rate was calculated. The differences in (early) implant failure rate were statistically tested (Fisher’s exact test).

## 3. Results

The follow up data of 1063 patients that received 2918 implants has been included in the chart review. The patients have been treated, *i.e.*, implants inserted between October 2007 and July 2012. 1337 (45.8%) of the reported implants had INICELL and 1581 (54.2%) TST enossal surfaces. As mentioned above, the hydrophilicity/hydrophobicity of the enossal surface was the only difference between both implant types.

**Figure 1:**
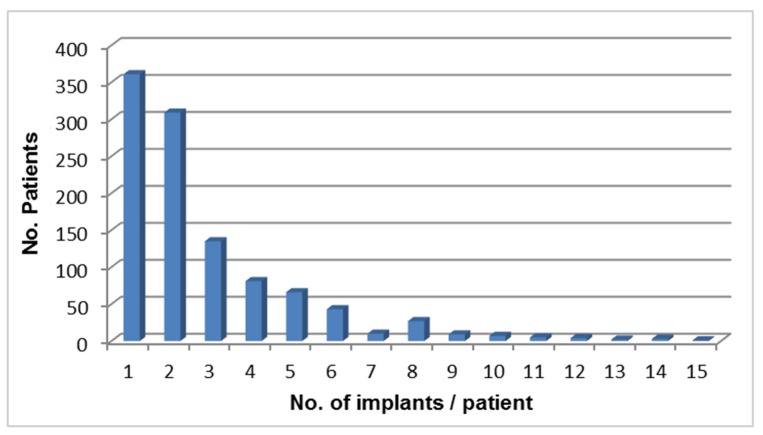
Number of implants/patient.

Thirty four percent of the patients have received 1 implant, 29% two, 13% three and 24% of patients received 3–15 (1 patient) implants (Figure 1), *i.e.*, most frequently placed were 1–2 implants per patient.

**Figure 2:**
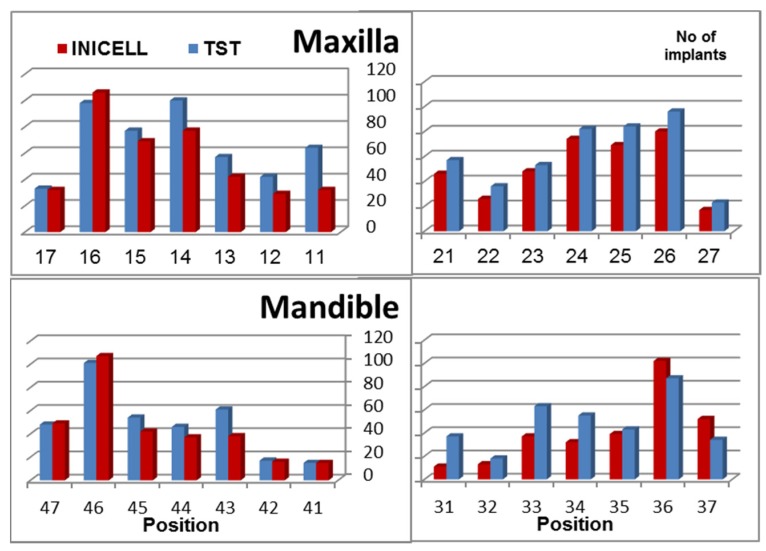
Number of INICELL and TST implants shown on the vertical axis is split by position in both jaws.

**Figure 3:**
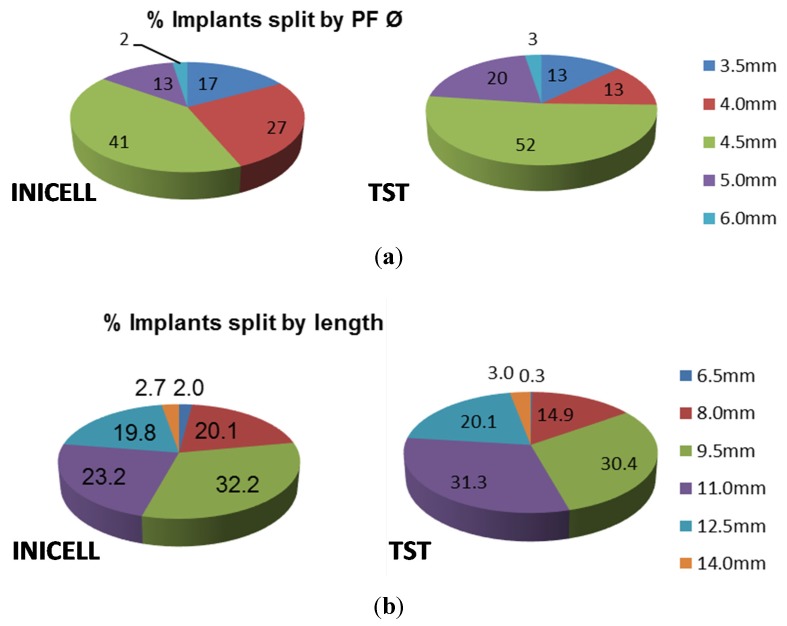
Overview of used implants by (**a**) diameter and (**b**) length.

Of the INICELL implants, 56% were inserted in the maxilla and 44% in the mandible. Similarly, 57% TST were placed in the upper and 43% in the lower jaw (Figure 2). The placement pattern between the INICELL and TST groups was quite similar, thereby validating the homogeneity of the presented sample. The replacement of individual tooth positions was balanced (Figure 2). Both INICELL and TST implants had platform diameters (PF Ø) 3.5–6.0 mm and they were 6.5–14.0 mm long (Figure 3).

Please note that 100% is the number of all included implants. The relative proportions were calculated for each platform diameter (PF Ø 3.5–6.0 mm) or implant length (6.5–14.0 mm).

Immediate loading was the case in 75 (7.1%) edentulous patients that have received 493 (16.9%) implants (267 (20%) INICELL and 226 (14.3%) TST). Immediate temporization was done in 74 (7.0%) patients. These patients received 35 (2.6%) INICELL and 39 (2.5%) TST implants. Bone grafting material was used with 258 (8.8%; INICELL and TST) implants. β calcium triphosphate (TCP, CEROS) was used in 122 (11.5%), whereas Bio-Oss in 136 (12.8%) cases. Both bone grafting materials were used mainly for simultaneous and two-stage sinus lifting (29 (2.7%) and 53 (5.4%) cases respectively), sinus lift according to Summers [6] (158 cases, *i.e.*, 14.9%) and guided bone regeneration (92 cases, *i.e.*, 8.7%). Autogenous bone was used in 36 (3.4%) cases, from the retromolar area and autologous bone (TBF, France) in 6 (0.6%) cases. In 56 (5.3%) cases an augmentation surgery has been performed prior to the implant placement. The bone healing time was 4 months when autogenous bone was used, 5 months when patients received allogeneic bone, and 6 months or more with the bovine bone.

The implant healing time recommendations of the manufacturer were also followed. Typically, it was 3 and 8 weeks for INICELL and 6 or 12 weeks for TST implants that were restored as single crowns. In edentulous patients immediate loading was done whenever possible, or temporary (non occlusal) crown was attached immediately after implantation (see above).

The average follow up time was 2.1 (1.1–5.4) years for INICELL and 4.5 (1.3–5.9) years for TST implants. In the reported period 7 (0.5%) implants with INICELL and 23 (1.5%) TST implants have failed. This difference is statistically significant (Table 1).

**Table 1:** Statistical comparison of the INICELL and TST survival rates (Fisher’s exact test; * *p* < 0.01).

No. of implants	Failed Implants	CSR	Observation Period
Total	30	99.0	4.0 (1.1–5.9) years
INICELL	7 *	99.5	2.1 (1.1–5.4) years
TST	23 *	98.5	4.5 (1.3–5.9) years

All implant failures occurred early, *i.e.*, before functional implant loading at the radiological check of the osseointegration at 2 months after implant placement. As implants have been loaded immediately, the check of integration was done at 2 months later by X-ray and clinically after removing the provisional crown. An overview of the failed implants is given in Table 2.

**Table 2:** Factors contributing to implant failure.

Contributing Factor	Number of Failed Implants
**INICELL: 7 failed implants**	
Reduced implant diameter (PF Ø 3.5 mm)	3
Immediate loading	2
Provisional loading	1
Edentulous patient with immediate loading	1
**TST: 23 failed implants**	
Reduced implant diameter (PF Ø 3.5 mm)	5
Edentulous patient with immediate loading	6 (in 4 patients)
Sinus lift	3 (in 2 patients)
Immediate temporization	9

The reported implant failures resulted in an overall cumulative survival rate of 99.0%. This is quite satisfactory considering the mean observation period of 4 (1.1–5.9) years. For INICELL the cumulative survival rate was 99.5%, for TST implants it was 98.5%. The apparently lower CSR of the TST implants (Table 1) was not attributable to the longer observation time of TST 4.5 (1.3–5.9) years as compared to INICELL implants 2.1 (1.1–5.4) years, as all of the failures occurred before implant loading. As for biological complications, no cases of mucositis or peri-implantitis were noticed in the observation period.

The lower early survival rate of INICELL implants was also mirrored in the survival rate recorded for individual indications:

- Standard cases: within the reported population 640 patients received 968 INICELL and 1064 TST implants. In standard cases implants have been placed after performing a full thickness flap and preparation of the implant bed by using the drilling sequence (recommended by the manufacturer). Most of the time a healing screw was placed and sutures done. 6 weeks later the permanent crown was fabricated and screw-attached with the torque recommended by the manufacturer (25 N for platforms between 4 and 6 mm and 15 N for the 3.5 mm). Early failures have occurred with only 1 INICELL and 9 TST implants. This difference was also statistically significant (*p* < 0.05; Fisher’s exact test).
- Edentulous cases: 75 edentulous patients received 493 immediately loaded implants (267 INICELL and 226 TST). Of these 2 INICELL and 6 TST implants have failed early. In some of these patients the implants have been placed with the help of navigated surgery [7].
- Immediate loading: immediate non-occlusal loading of single tooth was done in 74 patients with 35 INICELL and 39 TST implants. Only 1 INICELL implant failed early.
- Sinus floor elevation: Consecutive and simultaneous sinus lift was done in 240 patients that have received 86 INICELL and 141 TST implants. Only 3 TST implants failed early.
- Reduced diameter implants: from the 228 INICELL and 203 TST inserted reduced diameter (PF Ø 3.5 mm) implants early failures occurred with 5 TST and 3 INICELL implants (this difference is not statistically significant). This finding is in apparent contradiction to the outcome of a retrospective analysis of papers published until September 2012 [8]. In the presented study only a small proportion (13%) of the implants had a reduced diameter (3.5 mm). In this single center case series their survival rate was no different from that of wider diameter implants. Their outcome did not differ between INICELL or TST implants.

## 4. Discussion

Most recent studies suggested a low early failure rate of <1% and a success rate of >95% at 10 years for non-smokers [9]. Wang *et al.* [10] reported the clinical outcome of implants re-implanted into sites in after early failure. The newly placed implants were followed up for approximately 5.5 years. The obtained survival rate of 94.6% was quite favorable and so was the 90.6% success (optimum health) rate. Garcia-Bellostá *et al.* [11] reported the long term survival of 980 implants that were inserted in 323 patients a periodontal practice. After 5 years follow up the CSR was 96.2%. The authors were not able to detect any significant influence of smoking on implant survival. The reported CSR is also comparable with the results obtained in a similar retrospective evaluation of the TST implants [12]. The authors have compared the survival of TST implants with reduced and standard diameters. Their report comprised 332 patients which received 736 TST implants in three practices in Switzerland. The main finding was that the CSR of TST implants with reduced diameter (99.5%) was no different from the CSR of standard diameter implants (99.7%). The average follow up time was 20 months. Olate *et al.* [13] demonstrated that implant length and position in frontal regions correlated significantly with early failure rate. This finding was not confirmed in the presented study as the overall number of failed implants was quite low. The overall as well as individual INICELL and TST (early) failure rates reported in the presented retrospective analysis are therefore very well comparable with that of other implant systems.

The analysis of cases treated in a single implantological office for almost 6 years confirmed the very good clinical outcome that was achieved with both used implant lines. Only early failures have been observed with 7 INICELL and 23 TST implants. As a result of careful case evaluation, diagnosis and planning, a high overall survival rate of 99.0% was achieved after an average follow up period of 4 years. This compares well to other implant systems for which the survival rate was investigated in prospective clinical trials that usually exclude complex cases. It is also important to note that due to careful patient selection and care before surgery, no mucositis or peri-implantitis has occurred in this patient population within the observation period. Achieving such a high survival rate with no failures after implant loading is probably feasible only in a single center, skilled surgeon setting. Implant (early) failure rate is by far not the only clinically relevant treatment outcome. Patient-based outcomes are at least as important as the clinical and radiological parameters. They represent a significant complement to the assessment of the patient’s oral health status. An overview of the most frequently used patient-based outcome parameters are provided in this recently published review [14]. The optimal interplay of all components of the used implant line very likely contributed to the outstanding clinical outcome as well as survival rate. INICELL, the newly introduced hydrophilic enossal surface is very well suited for early loading [15]. It has also lead to significantly improved early failure rate. In spite of the limitations of this chart review analysis, the paper has achieved its goal, *i.e.*, the results of this quantitative long term assessment help to objectively assess the success rate of the implant treatment.

## 5. Conclusions

Within the limitations of this retrospective analysis, the overall early failure rate of the hydrophilic implants was significantly lower than that of hydrophobic implants. A large number of patients and implants has been followed long-term. The monitored implants had identical geometry, they differed only in the physico—chemical properties of their enossal surfaces, *i.e.*, hydrophilic (INICELL) and hydrophobic (TST) enossal surfaces have been compared. The use of hydrophilic implants allows the clinician to obtain less early failures, hence the interest of an up-to-date surface for the daily work of an implant office setting.
